# Identifying multimorbidity clusters in an unselected population of hospitalised patients

**DOI:** 10.1038/s41598-022-08690-3

**Published:** 2022-03-24

**Authors:** Lynn Robertson, Rute Vieira, Jessica Butler, Marjorie Johnston, Simon Sawhney, Corri Black

**Affiliations:** 1 Aberdeen Centre for Health Data Science, University of Aberdeen, Aberdeen, Scotland; 2 Institute of Applied Health Sciences, University of Aberdeen, Polwarth Building, Foresterhill, Aberdeen, AB25 2ZD Scotland; 3 Public Health Directorate, NHS Grampian, Aberdeen, Scotland

**Keywords:** Diseases, Health care, Medical research, Risk factors, Signs and symptoms

## Abstract

Multimorbidity (multiple coexisting chronic health conditions) is common and increasing worldwide, and makes care challenging for both patients and healthcare systems. To ensure care is patient-centred rather than specialty-centred, it is important to know which conditions commonly occur together and identify the corresponding patient profile. To date, no studies have described multimorbidity clusters within an unselected hospital population. Our aim was to identify and characterise multimorbidity clusters, in a large, unselected hospitalised patient population. Linked inpatient hospital episode data were used to identify adults admitted to hospital in Grampian, Scotland in 2014 who had ≥ 2 of 30 chronic conditions diagnosed in the 5 years prior. Cluster analysis (Gower distance and Partitioning around Medoids) was used to identify groups of patients with similar conditions. Clusters of conditions were defined based on clinical review and assessment of prevalence within patient groups and labelled according to the most prevalent condition. Patient profiles for each group were described by age, sex, admission type, deprivation and urban–rural area of residence. 11,389 of 41,545 hospitalised patients (27%) had ≥ 2 conditions. Ten clusters of conditions were identified: hypertension; asthma; alcohol misuse; chronic kidney disease and diabetes; chronic kidney disease; chronic pain; cancer; chronic heart failure; diabetes; hypothyroidism. Age ranged from 51 (alcohol misuse) to 79 (chronic heart failure). Women were a higher proportion in the chronic pain and hypothyroidism clusters. The proportion of patients from the most deprived quintile of the population ranged from 6% (hypertension) to 14% (alcohol misuse). Identifying clusters of conditions in hospital patients is a first step towards identifying opportunities to target patient-centred care towards people with unmet needs, leading to improved outcomes and increased efficiency. Here we have demonstrated the face validity of cluster analysis as an exploratory method for identifying clusters of conditions in hospitalised patients with multimorbidity.

## Introduction

Multimorbidity, defined as the coexistence of two or more conditions in the same individual, is common and increasing^1,2^. Patients with multimorbidity have poorer health outcomes and have greater use of acute unscheduled hospital healthcare^3^.

Patients with multimorbidity are heterogenous and can have a wide range of different combinations of conditions. Broad overall descriptions of health outcomes and needs of patients with multimorbidity, i.e. based on counts of conditions, are unhelpful for tailoring health care design. Accordingly, there have been recent calls to move away from simply counting diseases towards a more tailored understanding of which health conditions commonly co-occur^3,4^. This will enable us to anticipate the specific health needs of, and implications for, patients with particular conditions in combination.

Cluster analysis is a statistical technique that categorises items or properties into groups so that items in the same group are more statistically similar than those items in other groups. Our literature search highlighted that cluster analysis has previously been used to identify clusters of conditions in individuals from the general population, presenting in primary care, within narrow specialty subsets, or focussing specifically on older age groups^5^. We identified few studies that included hospitalised patients; three of which focussed on patients ≥ 65 years^6–8^, and one focussed on medical patients^9^. To our knowledge, no previous study has identified which conditions commonly cluster among unselected patients presenting to hospital and yet this is a setting of high strain on health systems globally.

As a first step to understanding the implications of disease clusters, we aimed to identify and characterise multimorbidity clusters in a cohort of patients hospitalised in the Grampian region of Scotland. This builds on our previous work describing the overall extent of hospital multimorbidity^10,11^.

## Methods

### Study design and setting

This study was prospectively preregistered on the Open Science Framework and is reported as per RECORD guidelines^12^. This is a population-based observational study using linked electronic health records carried out in a secondary care setting in a single health region in north-east Scotland (Grampian region, total population 2014, 584,220^13^). The region consists of one large urban centre and is spread over approximately 3000 square miles of city, town, village and rural communities^14^. Full details have been previously published^10,11^.

### Data sources

We used inpatient hospital episode data, namely the Scottish Morbidity Record (SMR)^15^, from general/acute (SMR01) and psychiatric (SMR04) admissions, from the years 2009–2014. SMR is an episode-based patient record relating to all patients discharged from hospital in Scotland. SMR data is collated in a national database, managed by Information Services Division Scotland^16^, and data is returned to each regional health authority on an ongoing basis. Data collected includes patient identifiable and demographic details, episode management details, general clinical information and death data. Clinical information is recorded as main diagnosis and up to five other significant diagnoses and coded using the World Health Organization’s International Classification of Diseases (ICD-10).

### Study population

**Figure 1:**
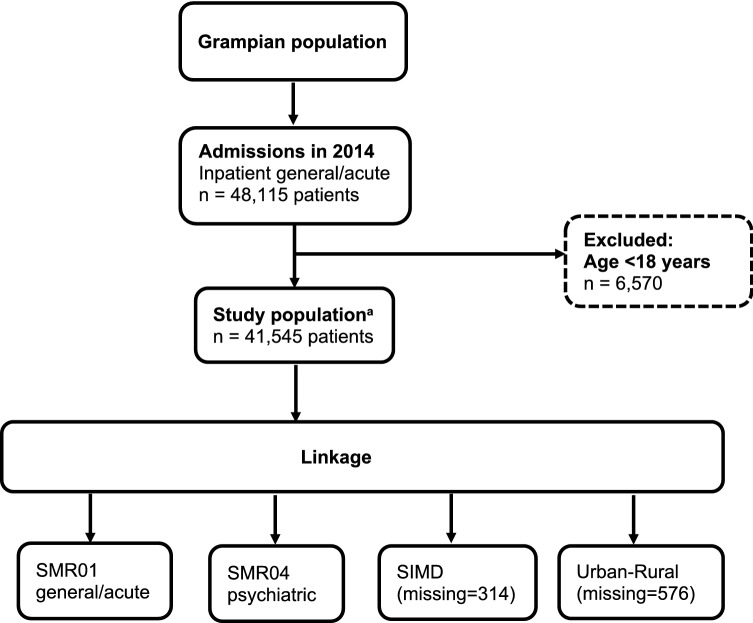
Flowchart of study population and data linkage. SMR, Scottish Morbidity Record; SIMD, Scottish Index of Multiple Deprivation. ^a^ Community Health Index number was missing or invalid for 662 inpatient general/acute admissions in 2014 (patients ≥ 18 years), therefore not included in the study population.

Adult patients (≥ 18 years) admitted to any hospital as an inpatient during 2014, in a single regional health authority (NHS Grampian) were included. A patient’s first admission in 2014 was classified as their “index admission”, and the admission date was classified as their “index date”. We excluded day case, obstetric and psychiatric admissions when identifying the study population. The flow diagram for identifying the study population is shown in Fig. 1. Patients with multimorbidity (≥ 2 conditions) were included in the present analysis (n = 11,389).

### Multimorbidity measure

Multimorbidity was defined as having recorded diagnoses of ≥ 2 chronic conditions^17,18^. Conditions were identified from general/acute and psychiatric admissions in the 5 years prior to index date. We used the multimorbidity measure developed by Tonelli et al.^19^. This was based on the measure developed by Barnett et al.^20^ for measuring multimorbidity in a primary care population, using coding unique to primary care in the UK^21^. Tonelli et al. developed a corresponding validated coding scheme for use with administrative data based on the ICD system^19^. The specific ICD-10 codes for the 30 conditions included are detailed in Additional file 1, with a note of minor amendments made. ICD-10 codes recorded as main or other diagnoses were included.

### Other variables

Other baseline characteristics were sex, age, deprivation, urban–rural area, and admission type. Age was categorised into six groups. Deprivation was measured using the Scottish Index of Multiple Deprivation (SIMD) 2012, categorised as quintiles (quintile 1 is the most deprived and quintile 5 the least deprived)^22^. Urban–rural status was measured using the Scottish Government sixfold Urban Rural Classification 2009/10^23^. SIMD and Urban Rural classification are identified from postcodes using the Scottish Government’s publicly available look-up files^24,25^.

### Data linkage

NHS Grampian SMR data were held in a dedicated secure server, managed by the accredited Grampian Data Safe Haven (DaSH)^26^. The Community Health Index (CHI) number, a unique patient identifier used throughout the Scottish health care system, was used to link the study population to hospital episode data by DaSH. Postcodes were used to link the study population to the SIMD and Urban Rural Classification. The de-identified dataset was prepared and hosted by the Grampian DaSH, allowing secure controlled access for researchers while ensuring data security.

There were 662 admissions with missing CHI numbers in 2014 (inpatient general/acute, ≥ 18 years), therefore these admissions were not included. There were 314 patients who could not be linked with SIMD, and 576 patients who could not be linked with Urban Rural Classification, because of missing or invalid postcodes (Fig. 1). The characteristics of patients with missing values are reported in Additional file 2.

### Statistical analysis

Descriptive analyses were expressed as frequencies and percentages or median and interquartile range (IQR) for categorical and continuous variables, respectively. Baseline characteristics were summarised by age, sex, admission type, SIMD quintile and Urban Rural category. The overall prevalence (%) was estimated for each condition and counts of conditions were calculated.

Clustering conditions, with each condition belonging exclusively to only one cluster, is a widely used approach to identify multimorbidity clusters. However, the same condition might occur in different combinations with other conditions in different patients. Patients with these different combinations of conditions, even if they share one same condition (e.g. Chronic Kidney Disease (CKD) and Diabetes, CKD and Chronic Heart Failure (CHF), only diabetes, only CKD, only CHF), might need a different plan of care.

An alternative valid clustering approach is to cluster patients instead, according to those combinations of conditions, which allows conditions to belong to more than one cluster of patients. While both methods are valid, we chose to cluster “patients” rather than “conditions”, as it better aligns with the purpose of identifying clusters of multimorbidity for improved person-centred care.

Relevant diagnosed chronic conditions in the previous 5 years were used to cluster patients with ≥ 2 conditions. Conditions were coded as binary variables, value of “1” when condition was present and “0” when absent. Prior to performing the cluster analysis, we evaluated whether the data contained non-random structures by visually inspecting the data (principle component analysis scatterplot Additional file 3) and using the Hopkins statistic^27^. These showed that the data was non-random, and therefore clusterable (Hopkins 0.28).

The Gower distance^28^ (equivalent to Jaccard^29^ when using only binary data) was used to measure the dissimilarity between observations. The Partitioning around Medoids (PAM) algorithm^30^ was used to identify distinct groups of patients with similar patterns of conditions, classifying individuals into mutually exclusive groups. The Silhouette method^31^ was used as an internal validation metric to determine the optimal number of patient groups, which was the number of groups that yielded the highest silhouette value. The groups were interpreted using descriptive statistics and the dimension reduction technique t-distributed stochastic neighbourhood embedding (t-SNE) was used to visualise the clusters^32^.

We carried out several sensitivity analyses. We compared the results obtained by: 1. replacing Gower with the Hamming distance^33^; 2. excluding the most common condition (hypertension) from the clustering process; and 3. excluding conditions with a prevalence of < 5% from the clustering process. Analyses were conducted using STATA v13.0 and R version 3.6.1.

### Defining clusters of conditions

Prior to analysis, we documented the clusters we would expect to observe. The patterns of conditions present in the resulting groups of patients were clinically reviewed by clinical members of the study group (CB, MJ, SS). Clusters of conditions within each patient group were defined based on a combination of clinical review and assessment of the highest prevalence conditions within each patient group and labelled according to the condition with the highest prevalence.

### Study registration

This study was prospectively pre-registered on the Open Science Framework on 26 September 2019 (https://osf.io/qnpw2). Deviations from the pre-registered protocol are documented in Additional file 4 and analysis R code is available in Additional file 5.

### Ethics approval and consent to participate

This study was approved by North Node Privacy Advisory Committee (NNPAC Ref No. 6/001/19). The remit of this Committee is to provide researchers with access to NHS patient/health data within NHS Grampian for research purposes via a streamlined approach that incorporates Sponsorship, Ethics, Caldicott & R&D. Informed consent was waived by North Node Privacy Advisory committee as this research falls within the conditions for processing personal data that is “necessary for the performance of a task carried out in the public interest or in the exercise of official authority vested in the controller; (Article 6 1,e of the UK General Data Protection Regulation (GDPR))”. Data was de-identified pre-analysis. All methods were performed in accordance with the relevant guidelines and regulations.

## Results

### Baseline characteristics

Of a total of 41,545 patients hospitalised in 2014, 11,389 (27.4%) had multimorbidity (≥ 2 conditions). Table 1 shows that patients with multimorbidity were older and more frequently admitted as an emergency than those with < 2 conditions. Just over half of patients were from the two least deprived quintiles. Counts of conditions in patients with multimorbidity ranged from 2 to 11, and the most common conditions were hypertension (56.5%), diabetes (27.0%), chronic kidney disease (26.0%), atrial fibrillation (19.9%) and chronic pulmonary disease (18.6%) (Table 2). The least common conditions were multiple sclerosis (1.1%), schizophrenia (0.8%), psoriasis (0.7%), peripheral vascular disease (0.2%) and chronic viral hepatitis B (0.1%).

**Table 1:** Baseline characteristics and counts of conditions.

	< 2 Conditions	≥ 2 Conditions
	n (%)	n (%)
Total number of patients	30,156	11,389
**Sex**		
Male	14,354 (47.6)	5323 (46.7)
Female	15,802 (52.4)	6066 (53.3)
**Median age (IQR)**	56 (39–71)	73 (61–81)
**Age groups**		
18–29	4400 (14.6)	277 (2.4)
30–44	5277 (17.5)	655 (5.8)
45–59	7023 (23.3)	1648 (14.5)
60–74	7499 (24.9)	3661 (32.1)
75–89	5259 (17.4)	4446 (39.0)
≥ 90	698 (2.3)	702 (6.2)
**Admission type**		
Routine	9867 (32.7)	2887 (25.3)
Emergency	20,289 (67.3)	8502 (74.7)
**SIMD 2012**^**a**^		
1 (most deprived)	2364 (7.8)	953 (8.4)
2	4443 (14.7)	1836 (16.1)
3	7047 (23.4)	2839 (24.9)
4	7919 (26.3)	2873 (25.2)
5 (least deprived)	8104 (26.9)	2853 (25.1)
**Urban rural**^**a**^		
Large urban	11,089 (36.8)	4488 (39.4)
Other urban	4488 (14.9)	1841 (16.2)
Accessible small town	2404 (8.0)	925 (8.1)
Remote small town	2667 (8.8)	1052 (9.2)
Accessible rural	5488 (18.2)	1996 (17.5)
Remote rural	3532 (11.7)	999 (8.8)
**Number of conditions**		
0	22,884 (75.9)	0
1	7272 (24.1)	0
2	0	5173 (45.4)
3	0	3241 (28.5)
4	0	1665 (14.6)
5	0	783 (6.9)
6	0	357 (3.1)
7	0	100 (0.9)
8	0	56 (0.5)
9	0	9 (0.1)
10 + ^b^	0	5 (0.0)

IQR, inter-quartile range; SIMD, Scottish Index of Multiple Deprivation.

^a^314 patients had missing values for SIMD category (< 2 n = 279, ≥ 2 n = 35) and 576 patients had missing values for Urban Rural category (< 2 n = 488, ≥ 2 n = 88).

^b^Rows reporting number of patients with 10 and 11 conditions have been collapsed due to counts < 5.

**Table 2:** Prevalence of individual conditions among patients with multimorbidity.

Condition	n (%)	
Total number of patients	11,389	
Hypertension	6430	(56.5)
Diabetes	3071	(27.0)
Chronic kidney disease	2959	(26.0)
Atrial fibrillation and flutter	2262	(19.9)
Chronic pulmonary disease (excludes asthma)	2115	(18.6)
Asthma	2074	(18.2)
Chronic pain	1937	(17.0)
Hypothyroidism	1647	(14.5)
Chronic heart failure	1470	(12.9)
Cancer, non-metastatic (breast, cervical, colorectal, lung, prostate)	1291	(11.3)
Severe constipation	1168	(10.3)
Alcohol misuse	1155	(10.1)
Myocardial infarction	881	(7.7)
Depression	786	(6.9)
Stroke or transient ischaemic attack	764	(6.7)
Cancer, metastatic	635	(5.6)
Dementia	586	(5.1)
Rheumatoid arthritis	579	(5.1)
Epilepsy	432	(3.8)
Cirrhosis (and hepatic decompensation)	383	(3.4)
Inflammatory bowel disease	347	(3.0)
Peptic ulcer disease (excluding bleeding)	214	(1.9)
Parkinson's disease	188	(1.7)
Cancer, lymphoma	179	(1.6)
Irritable bowel syndrome	170	(1.5)
Multiple sclerosis	128	(1.1)
Schizophrenia	89	(0.8)
Psoriasis	83	(0.7)
Peripheral vascular disease	27	(0.2)
Chronic viral hepatitis B	16	(0.1)

### Multimorbidity clusters

Cluster analysis of disease occurrence identified ten groups of patients. Table 3 describes the prevalence of all conditions in each patient group. Within each patient group, clusters of conditions have been highlighted in bold and labelled according to the most prevalent condition in each group. Other conditions that featured in a patient group (i.e. less common conditions with a higher prevalence than in other groups) are highlighted in italics.

**Table 3:** Prevalence of 30 conditions by patient group.

Group number	Total		1	2	3	4	5	6	7	8	9	10
Cluster label			HTN	Asthma	Alcohol	CKD/diabetes	CKD	Pain	Cancer	CHF	Diabetes	Hypo
Number of patients	**11,389**		**2590**	**1290**	**931**	**878**	**1396**	**834**	**654**	**694**	**1614**	**508**
Prevalence of conditions	n	%	%	%	%	%	%	%	%	%	%	%
Alcohol misuse	1155	10.1	6.9	6.0	**75.4**	3.4	4.1	2.0	0.9	3.2	3.4	1.8
Asthma	2074	18.2	4.7	**68.9**	*20.5*	12.2	11.3	*20.6*	8.6	9.5	14.1	17.1
Atrial fibrillation and flutter	2262	19.9	**59.0**	5.4	0.4	*17.8*	0.0	0.0	1.7	**71.2**	0.0	0.0
Cancer, lymphoma	179	1.6	1.9	1.8	0.8	1.6	1.6	2.0	1.2	1.9	1.1	2.0
Cancer, metastatic	635	5.6	2.3	0.7	0.5	1.7	2.7	0.7	**71.7**	0.9	1.7	0.4
Cancer, non-metastatic	1291	11.3	*10.5*	5.7	3.7	4.8	8.0	2.2	**92.0**	4.3	5.9	2.8
Chronic heart failure	1470	12.9	*11.7*	5.7	3.9	*17.4*	6.7	5.5	2.1	**84.6**	8.7	4.9
Chronic kidney disease	2959	26.0	8.2	5.2	1.2	**100.0**	**100.0**	0.0	4.0	**53.2**	0.0	0.0
Chronic pain	1937	17.0	6.5	8.4	15.7	12.8	16.8	**100.0**	10.2	8.1	13.1	0.0
Chronic pulmonary disease (excludes asthma)	2115	18.6	8.6	**68.4**	13.0	15.0	10.9	12.1	12.5	*25.8*	10.3	14.8
Chronic viral hepatitis B	16	0.1	0.2	0.0	0.5	0.0	0.1	0.2	0.0	0.0	0.1	0.0
Cirrhosis and hepatic decompensation	383	3.4	2.4	2.7	*12.1*	3.9	2.6	0.8	3.2	2.4	2.9	2.0
Dementia	586	5.1	8.6	2.8	3.7	4.4	6.1	4.4	0.6	5.9	3.2	6.9
Depression	786	6.9	3.1	3.9	**54.6**	3.5	3.9	1.4	0.5	1.6	1.5	2.4
Diabetes	3071	27.0	11.0	5.5	*15.1*	**100.0**	0.0	0.0	3.2	8.9	**100.0**	0.0
Epilepsy	432	3.8	4.0	5.2	*9.9*	1.8	3.1	2.9	2.9	1.3	1.9	5.5
Hypertension	6430	56.5	**77.5**	**58.1**	6.3	**72.2**	**64.7**	**53.7**	9.9	14.7	**71.4**	**60.8**
Hypothyroidism	1647	14.5	7.7	5.5	9.6	12.0	14.7	14.9	7.8	10.2	13.8	**100.0**
Inflammatory bowel disease	347	3.0	4.1	4.0	1.5	1.9	3.5	3.7	2.9	1.3	1.9	3.9
Irritable bowel syndrome	170	1.5	1.5	2.2	2.4	0.6	0.9	3.6	0.6	0.4	0.9	2.0
Multiple sclerosis	128	1.1	1.6	0.9	0.9	0.6	1.1	1.2	0.9	0.1	1.5	1.0
Myocardial infarction	881	7.7	10.0	6.5	2.6	11.7	5.5	4.3	2.1	*21.3*	6.8	5.3
Parkinson's disease	188	1.7	2.7	0.9	1.4	0.7	1.6	2.3	0.5	1.9	1.4	1.0
Peptic ulcer disease excluding bleeding	214	1.9	2.4	1.7	3.2	1.1	1.5	2.2	0.8	1.6	1.7	1.2
Peripheral vascular disease	27	0.2	0.2	0.3	0.0	0.3	0.3	0.1	0.0	0.3	0.3	0.4
Psoriasis	83	0.7	0.5	0.5	1.2	0.5	1.1	1.3	0.5	0.3	0.7	1.2
Rheumatoid arthritis	579	5.1	5.5	6.3	1.6	4.0	7.3	8.6	3.2	4.0	3.0	6.7
Schizophrenia	89	0.8	0.4	1.0	*4.0*	0.2	0.6	0.6	0.3	0.1	0.4	0.6
Severe constipation	1168	10.3	11.2	10.9	11.0	9.3	10.6	12.2	13.3	7.2	6.8	11.0
Stroke or transient ischaemic attack	764	6.7	12.6	5.3	4.5	5.7	5.4	4.1	2.1	6.3	5.5	3.9

HTN, hypertension; CKD, chronic kidney disease; CHF, chronic heart failure; hypo, hypothyroidism.

Note: Values in bold represent clusters of conditions within patient groups i.e. the highest prevalence conditions within patient groups. Values in italics represent other conditions that feature in a patient group i.e. less common conditions with a higher prevalence than in other groups.

For example, Group 1 (labelled “hypertension”) was characterised by hypertension (77.5%) and atrial fibrillation (59.0%). Other feature conditions were non-metastatic cancer and chronic heart failure. Group 3 (“alcohol misuse”) was characterised by alcohol misuse (75.4%) and depression (54.5%). Other feature conditions in this group were asthma, cirrhosis, diabetes, epilepsy and schizophrenia.

The number of patients in each group ranged from 508 (hypothyroidism) to 2590 (hypertension). Several conditions were present in multiple groups of patients. Seven of the ten groups included hypertension, three included chronic kidney disease, two diabetes, and two atrial fibrillation. Multimorbidity clusters are summarised in Table 4.

**Table 4:** Multimorbidity clusters.

Cluster label	Most prevalent conditions
Hypertension	Hypertension, atrial fibrillation and flutter
Asthma	Asthma, chronic pulmonary disease, hypertension
Alcohol misuse	Alcohol misuse, depression
Chronic kidney disease/diabetes	Chronic kidney disease, diabetes, hypertension
Chronic kidney disease	Chronic kidney disease, hypertension
Chronic pain	Chronic pain, hypertension
Cancer	Metastatic cancer, non-metastatic cancer
Chronic heart failure	Atrial fibrillation, chronic heart failure, chronic kidney disease
Diabetes	Diabetes, hypertension
Hypothyroidism	Hypertension, hypothyroidism

Table 5 describes the characteristics of patients in each group. Median age ranged from 51 (Group 3 alcohol misuse) to 79 (Group 8 chronic heart failure) years. The groups with the highest proportion of females were Group 6 (chronic pain), and Group 10 (hypothyroidism). The groups with the highest proportion of males were Group 3 (alcohol misuse), and Group 8 (chronic heart failure). The proportion of patients from the most deprived quintile ranged from 6.3% to 14.1%. Group 3 (alcohol misuse) had the highest proportion of patients from the most deprived and large urban areas, while Group 7 (cancer) had the highest proportion of patients from the least deprived and rural areas. Median counts of conditions ranged from 2 to 4, with Group 4 (CKD/diabetes) and Group 8 (chronic heart failure) having the highest proportion of patients with five or more conditions. The highest proportion of patients admitted as an emergency was in Group 3 (alcohol misuse) and Group 8 (chronic heart failure).

**Table 5:** Characteristics by patient group.

Group number	Total		1	2	3	4	5	6	7	8	9	10
Cluster label			HTN	Asthma	Alcohol	CKD/diabetes	CKD	Pain	Cancer	CHF	Diabetes	Hypo
Number of patients	11,389		2590	1290	931	878	1396	834	654	694	1614	508
	n	%	%	%	%	%	%	%	%	%	%	%
**Median age (IQR)**	73 (61–81)		78 (68–84)	70 (60–79)	51 (39–64)	75 (66–81)	76 (64–84)	69 (56–79)	66 (56–74)	79 (70–85)	71 (62–79)	74 (64–82)
**Sex**												
Male	5323	46.7	51.6	42.3	53.1	52.7	42.0	35.4	44.6	58.9	50.7	15.9
Female	6066	53.3	48.4	57.7	46.9	47.3	58.0	64.6	55.4	41.1	49.3	84.1
**SIMD**^**a**^												
1 (most deprived)	953	8.4	6.3	11.9	14.1	8.1	8.7	9.1	6.9	6.8	6.9	6.9
2	1836	16.1	14.5	17.5	21.2	16.7	14.9	15.9	11.6	15.4	17.0	17.9
3	2839	24.9	24.7	28.1	27.0	26.3	24.2	21.7	21.3	24.2	26.0	21.5
4	2873	25.2	26.9	22.3	19.3	25.6	25.3	25.2	28.0	25.9	26.5	25.8
5 (least deprived)	2853	25.1	27.4	19.7	18.0	23.1	26.4	27.7	32.1	27.4	23.3	28.0
**Urban rural**^**a**^												
Large urban	4488	39.4	38.4	41.3	49.4	35.8	39.8	39.7	36.4	40.3	35.6	40.9
Other urban	1841	16.2	15.4	15.9	15.9	16.9	17.3	15.6	14.8	17.7	16.5	16.5
Accessible small town	925	8.1	8.3	8.1	6.1	8.1	8.7	9.7	8.3	8.1	8.1	7.1
Remote small town	1052	9.2	9.8	8.5	8.6	10.3	9.8	7.8	8.4	8.9	9.1	10.2
Accessible rural	1996	17.5	17.5	18.1	13.3	19.9	14.7	17.3	20.3	16.0	20.8	16.3
Remote rural	999	8.8	10.1	7.1	5.5	8.4	8.7	9.0	10.6	8.6	9.2	8.9
**Number of conditions**												
Median count (IQR)	3 (2–4)		3 (2–3)	3 (2–4)	2 (2–3)	4 (3–5)	3 (2–4)	2 (2–3)	2 (2–3)	3 (2–4)	3 (2–3)	2 (2–3)
2	5173	45.4	49.8	48.6	51.1	9.9	43.2	54.8	59.0	30.1	46.3	57.3
3	3241	28.5	26.9	24.8	27.5	26.9	31.1	28.8	27.4	27.5	33.3	29.5
4	1665	14.6	13.6	13.8	13.9	26.0	15.6	11.3	9.3	19.5	13.5	10.0
5 +	1310	11.5	9.7	12.8	7.5	37.2	10.1	5.2	4.3	22.9	6.9	3.1
**Admission type**												
Routine	2887	25.3	23.4	22.2	13.7	26.1	25.6	35.7	33.8	15.6	30.5	31.5
Emergency	8502	74.7	76.6	77.8	86.3	73.9	74.4	64.3	66.2	84.4	69.5	68.5

HTN, hypertension; CKD, chronic kidney disease; CHF, chronic heart failure; hypo, hypothyroidism; IQR, inter-quartile range; SIMD, Scottish Index of Multiple Deprivation.

^a^35 patients had missing values for SIMD category and 88 patients had missing values for Urban Rural category.

### Sensitivity analyses

Results of the three sensitivity analyses are shown in Additional file 6. The sensitivity analyses using the Hamming distance or excluding hypertension resulted in similar clusters being identified. Excluding conditions with a prevalence of < 5%, resulted in 13 clusters, with some clusters split over more smaller groups compared with the main analysis. For example, the asthma and chronic pulmonary disease cluster was split into two separate clusters. However, overall, the same conditions were identified.

## Discussion

To our knowledge, this is the first study to describe multimorbidity clusters in an unselected inpatient adult population, and the first population-level study of multimorbidity clusters in a Scottish/UK hospitalised population. Of 41,545 patients admitted to hospital, approximately one quarter (11,389) had multimorbidity, and our analysis identified ten clusters of co-occurring conditions.

The clusters revealed recognisable co-occurrences where the link was potentially causal e.g. hypertension leading to atrial fibrillation^34^, diabetes leading to kidney disease^35^. Clusters also revealed shared underlying disease mechanisms. For example, chronic heart failure, myocardial infarction, atrial fibrillation, stroke and kidney disease as vascular conditions of older age^36^. We identified a group of patients with a high prevalence of alcohol misuse co-occurring with depression and asthma, predominantly male and from more deprived quintiles, possibly indicating an underlying social driver. This finding supports known inequalities in alcohol-attributable harms, given that disadvantaged social groups have greater alcohol-attributable harms (admissions or death) compared with more advantaged individuals^37^. There were also clusters that represented artefact of how conditions are classified e.g. metastatic disease with non-metastatic cancer as two conditions in one person. Conditions with a high prevalence also had an impact, for example hypertension was present in more than half of those with multimorbidity and was a key condition in seven out of ten clusters.

While these clusters have face validity, their usefulness depends upon how they might delineate groups of people with specific health and social needs. Relevantly, those in the chronic heart failure cluster were the oldest (median age 79), and more likely to have 5 + health conditions; whereas those in the alcohol misuse cluster were more likely to be of working age (median age 51), live in a deprived area and present to hospital as an emergency. Thus, notwithstanding the artefact of associations between very common conditions, e.g. hypertension, and those that are prerequisites of another, e.g. metastatic cancer, we have shown the potential of identifying key multimorbidity clusters to which people may belong, so that we can ensure that health and social support is prioritised to the inpatient areas.

Methodological heterogeneity in studies investigating multimorbidity clusters makes it difficult to make comparisons. Studies vary with regard to the number and type of conditions included, data sources, populations, settings, and clustering methods. The most comparable study, in adult medical inpatients, identified five clusters of conditions: neurological diseases, heart/kidney diseases, malignancy, psychiatric diseases and miscellaneous diseases, from a list of 17 conditions^9^. We also identified similar chronic heart failure and cancer clusters.

This was a large, population-based study, and to our knowledge, the first study to characterise patterns of multimorbidity in an unselected hospitalised population. We ascertained conditions over the 5 years prior to index date, as longer lookback periods are more effective for identifying conditions^38,39^. We used high quality administrative data, with quality assurance assessments undertaken to ensure that inpatient data items were being recorded consistently and to a high standard^40^. Our results should be generalisable to other hospitalised populations with similar characteristics, and furthermore, the methodology used in this study would be applicable to health systems worldwide.

Limitations should also be noted. Cluster analysis is an exploratory classification method, and different clustering algorithms may produce different results. We found that hierarchical cluster analysis did not produce clinically relevant clusters, and therefore have reported results from PAM. To help with this, sensitivity analyses were conducted, the final clustering solution was clinically reviewed, and we have transparently and comprehensively reported our methods and deviations from pre-registered protocol (Additional file 4). Another limitation was that as conditions were identified from hospital episode data in the 5 years prior to index admission, we will not have recorded conditions for patients who were first time presenters on the index date. We did not include conditions from primary care records which will have underestimated the multimorbidity burden among people with conditions predominantly looked after in primary care. However, it is reasonable to hypothesise that conditions which are rare in the hospital setting, would have less influence on health care needs of people in hospital. Finally, multimorbidity clustering was based specifically on the conditions in Tonelli’s measure of multimorbidity^19^. There are many other heterogenous measures of multimorbidity available and we acknowledge that our findings may change if other conditions are studied. However, there is no single recommended measure of multimorbidity available. Therefore, we selected Tonelli as it is a validated adaptation of the highly influential Barnett measure.

The value of identifying clusters of conditions in hospitalised patients is as a first step towards identifying opportunities to target patient-centred care towards people with unmet needs. An important next step will be to determine the clinical outcomes of patients in each cluster, reasons why patients within some clusters have poor outcomes, and the pathways through healthcare that patients in each cluster predominantly take.

## Conclusions

Identifying clusters of conditions in hospital patients is a first step towards identifying opportunities to target patient-centred care towards people with unmet needs, leading to improved outcomes and increased efficiency. Here we have demonstrated the face validity of cluster analysis as an exploratory method for identifying clusters of conditions in hospitalised patients with multimorbidity.

## Supplementary Information

Supplementary Information 1.

Supplementary Information 2.

Supplementary Information 3.

Supplementary Information 4.

Supplementary Information 5.

Supplementary Information 6.

## Data Availability

The data that support the findings of this study are available in the Grampian Data Safe Haven [Dash140/DaSH326], provided the necessary permissions have been obtained. Further information is available at http://www.abdn.ac.uk/iahs/facilities/grampian-data-safe-haven.php and requests for data may be made to Professor Corri Black on behalf of Grampian Data Safe Haven, corri.black@abdn.ac.uk.

## Abbreviations

SMR: Scottish Morbidity Record
ICD: International Classification of Diseases
SIMD: Scottish Index of Multiple Deprivation
DaSH: Data Save Haven
CHI: Community Health Index
IQR: Interquartile range
PAM: Partitioning around Medoids
t-SNE: T-distributed stochastic neighbourhood embedding
