# Supplementary material for: Identifying multimorbidity clusters in an unselected population of hospitalised patients
Source: Sci Rep. 2022 Mar 24;12:5134. doi: 10.1038/s41598-022-08690-3 (PMC8948299; doi:10.1038/s41598-022-08690-3)
Supplement: Supplementary file 1 — Supplementary Information 1. [file 41598_2022_8690_MOESM1_ESM.pdf]

Additional file 1. Coding definitions (ICD-10) for 30 conditions included in Tonelli et al. (2015)

Condition	ICD-10 codes	Description
Alcohol misuse	E52	Niacin deficiency
	F10	Mental and behavioural disorders due to use of alcohol
	G62.1	Alcoholic polyneuropathy
	I42.6	Alcoholic cardiomyopathy
	K29.2	Alcoholic gastritis
	K70.0	Alcoholic fatty liver
	K70.3	Alcoholic cirrhosis of liver
	K70.9	Alcoholic liver disease, unspecified
	T51	Toxic effect of alcohol
	Z50.2	Alcohol rehabilitation
	Z71.4	Alcohol abuse counselling and surveillance
	Z72.1	Alcohol use
Asthma	J45	Asthma
Atrial fibrillation	I48	Atrial fibrillation and flutter
Cancer, lymphoma	C81	Hodgkin lymphoma
	C82	Follicular lymphoma
	C83	Non-follicular lymphoma
	C84	Mature T/NK-cell lymphomas
	C85	Other and unspecified types of non-Hodgkin lymphoma
	C88	Malignant immunoproliferative diseases
	C90.0	Multiple myeloma
	C90.2	Extramedullary plasmacytoma
	C96	Other leukaemias of specified cell type
Cancer, metastatic	C77	Secondary and unspecified malignant neoplasm of lymph nodes
	C78	Secondary malignant neoplasm of respiratory and digestive organs
	C79	Secondary malignant neoplasm of other and unspecified sites
	C80	Malignant neoplasm, without specification of site
Cancer, non-metastatic (breast, cervical, colorectal, lung, prostate)	C18	Malignant neoplasm of colon
	C19	Malignant neoplasm of rectosigmoid junction
	C20	Malignant neoplasm of rectum
	C21	Malignant neoplasm of anus and anal canal
	C33	Malignant neoplasm of trachea
	C34	Malignant neoplasm of bronchus and lung
	C38.4	Pleura
	C45.0	Mesothelioma of pleura
	C46.7	Kaposi sarcoma of other sites
	C50	Malignant neoplasm of breast
	C53	Malignant neoplasm of cervix uteri
	C61	Malignant neoplasm of prostate
	D01.0	Carcinoma in situ of Colon
	D01.1	Carcinoma in situ of Rectosigmoid junction
	D01.2	Carcinoma in situ of Rectum
	D01.3	Carcinoma in situ of Anus and anal canal
	D02.2	Carcinoma in situ of Bronchus and lung
	D05	Carcinoma in situ of breast
	D06	Carcinoma in situ of cervix uteri
	D07.5	Carcinoma in situ of Prostate
Chronic heart failure	I09.9	Rheumatic heart disease, unspecified
	I25.5	Ischaemic cardiomyopathy
	I42.0	Dilated cardiomyopathy
	I42.5	Other restrictive cardiomyopathy
	I42.6	Alcoholic cardiomyopathy
	I42.7	Cardiomyopathy due to drugs and other external agents
	I42.8	Other cardiomyopathies
	I42.9	Cardiomyopathy, unspecified
	I43	Cardiomyopathy in diseases classified elsewhere

Condition	ICD-10 codes	Description
	I50	Heart failure
Chronic kidney disease	N00-N23	Glomerular diseases (N00-N08)
		Renal tubulo-interstitial diseases (N10-N16)
		Renal failure (N17-N19)
		Urolithiasis (N20-N23)
Chronic pain	F45.4	Persistent somatoform pain disorder
	M08.1	Juvenile ankylosing spondylitis
	M25.5	Pain in joint
	M43.2	Other fusion of spine
	M43.3	Recurrent atlantoaxial subluxation with myelopathy
	M43.4	Other recurrent atlantoaxial subluxation
	M43.5	Other recurrent vertebral subluxation
	M43.6	Torticollis
	M45	Ankylosing spondylitis
	M46.1	Sacroiliitis, not elsewhere classified
	M46.3	Infection of intervertebral disc (pyogenic)
	M46.4	Discitis, unspecified
	M46.9	Inflammatory spondylopathy, unspecified
	M47	Spondylosis
	M48.0	Spinal stenosis
	M48.1	Ankylosing hyperostosis [Forestier]
	M48.8	Other specified spondylopathies
	M48.9	Spondylopathy, unspecified
	M50.8	Other cervical disc disorders
	M50.9	Cervical disc disorder, unspecified
	M51	Other intervertebral disc disorders
	M53.1	Cervicobrachial syndrome
	M53.2	Spinal instabilities
	M53.3	Sacrococcygeal disorders, not elsewhere classified
	M53.8	Other specified dorsopathies
	M53.9	Dorsopathy, unspecified
	M54	Dorsalgia
	M60.8	Other myositis
	M60.9	Myositis, unspecified
	M63.3	Myositis in sarcoidosis
	M79.0	Rheumatism, unspecified
	M79.1	Myalgia
	M79.2	Neuralgia and neuritis, unspecified
	M79.6	Pain in limb
	M79.7	Fibromyalgia
	M96.1	Postlaminectomy syndrome, not elsewhere classified
Chronic pulmonary disease	I27.8	Other specified pulmonary heart diseases
	I27.9	Pulmonary heart disease, unspecified
	J40	Bronchitis, not specified as acute or chronic
	J41	Simple and mucopurulent chronic bronchitis
	J42	Unspecified chronic bronchitis
	J43	Emphysema
	J44	Other chronic obstructive pulmonary disease
	J46	Status asthmaticus
	J47	Bronchiectasis
	J60	Coalworker pneumoconiosis
	J61	Pneumoconiosis due to asbestos and other mineral fibres
	J62	Pneumoconiosis due to dust containing silica
	J63	Pneumoconiosis due to other inorganic dusts
	J64	Unspecified pneumoconiosis
	J65	Pneumoconiosis associated with tuberculosis
	J66	Airway disease due to specific organic dust
	J67	Hypersensitivity pneumonitis due to organic dust

Condition	ICD-10 codes	Description
	J68.4	Chronic respiratory conditions due to chemicals, gases, fumes and vapours
	J70.1	Chronic and other pulmonary manifestations due to radiation
	J70.3	Chronic drug-induced interstitial lung disorders
Chronic viral hepatitis B	B16	Acute hepatitis B
	B18.0	Chronic viral hepatitis B with delta-agent
	B18.1	Chronic viral hepatitis B without delta-agent
Cirrhosis and hepatic decompensation	K70.3	Alcoholic cirrhosis of liver
	K74.3	Primary biliary cirrhosis
	K74.4	Secondary biliary cirrhosis
	K74.5	Biliary cirrhosis, unspecified
	K74.6	Other and unspecified cirrhosis of liver
	I85.0	Oesophageal varices with bleeding
	I85.9	Oesophageal varices without bleeding
	I98.2	Oesophageal varices without bleeding in diseases classified elsewhere
	I98.3	Oesophageal varices with bleeding in diseases classified elsewhere
	K65.0	Acute peritonitis
	K65.8	Other peritonitis
	K65.9	Peritonitis, unspecified
	K67.0	Chlamydial peritonitis
	K67.1	Gonococcal peritonitis
	K67.2	Syphilitic peritonitis
	K67.3	Tuberculous peritonitis
	K67.8	Other disorders of peritoneum in infectious diseases classified elsewhere
	K76.7	Hepatorenal syndrome
	K93.0	Tuberculous disorders of intestines, peritoneum and mesenteric glands
	R18	Ascites
Dementia	F00	Dementia in Alzheimer disease
	F01	Vascular dementia
	F02	Dementia in other diseases classified elsewhere
	F03	Unspecified dementia
	F05.1	Delirium superimposed on dementia
	G30	Alzheimer disease
	G31.1	Senile degeneration of brain, not elsewhere classified
Depression	F20.4	Post-schizophrenic depression
	F31.3	Bipolar affective disorder, current episode mild or moderate depression
	F31.4	Bipolar affective disorder, current episode severe depression without psychotic symptoms
	F31.5	Bipolar affective disorder, current episode severe depression with psychotic symptoms
	F32	Depressive episode
	F33	Recurrent depressive disorder
	F34.1	Dysthymia
	F41.2	Mixed anxiety and depressive disorder
	F43.2	Adjustment disorders
Diabetes	E10	Insulin-dependent diabetes mellitus
	E11	Non-insulin-dependent diabetes mellitus
	E12	Malnutrition-related diabetes mellitus
	E13	Other specified diabetes mellitus
	E14	Unspecified diabetes mellitus
Epilepsy	G40	Epilepsy
	G41	Status epilepticus
Hypertension	I10	Essential (primary) hypertension
	I11	Hypertensive heart disease

Condition	ICD-10 codes	Description
	I12	Hypertensive renal disease
	I13	Hypertensive heart and renal disease
	I15	Secondary hypertension
Hypothyroidism	E00	Congenital iodine-deficiency syndrome
	E01	Iodine-deficiency-related thyroid disorders and allied conditions
	E02	Subclinical iodine-deficiency hypothyroidism
	E03	Other hypothyroidism
	E89.0	Postprocedural hypothyroidism
Inflammatory bowel disease	K50	Crohn disease [regional enteritis]
	K51	Ulcerative colitis
Irritable bowel syndrome	K58	Irritable bowel syndrome
Multiple sclerosis	G35	Multiple sclerosis
	G36	Other acute disseminated demyelination
	G37	Other demyelinating diseases of central nervous system
	H46	Optic neuritis
Myocardial infarction	I21	Acute myocardial infarction
	I22	Subsequent myocardial infarction
Parkinson's disease	G20	Parkinson disease
	G21	Secondary parkinsonism
	G22	Parkinsonism in diseases classified elsewhere
Peptic ulcer disease without haemorrhage	K25.7	Gastric ulcer-Chronic without haemorrhage or perforation
	K25.9	Gastric ulcer-Unspecified as acute or chronic, without haemorrhage or perforation
	K26.7	Duodenal ulcer-Chronic without haemorrhage or perforation
	K26.9	Duodenal ulcer-Unspecified as acute or chronic, without haemorrhage or perforation
	K27.7	Peptic ulcer, site unspecified- Chronic without haemorrhage or perforation
	K27.9	Peptic ulcer, site unspecified- Unspecified as acute or chronic, without haemorrhage or perforation
	K28.7	Gastrojejunal ulcer- Chronic without haemorrhage or perforation
	K28.9	Gastrojejunal ulcer- Unspecified as acute or chronic, without haemorrhage or perforation
Peripheral vascular disease	I70.2	Atherosclerosis of arteries of extremities
Psoriasis	L40.0	Psoriasis vulgaris
	L40.1	Generalized pustular psoriasis
	L40.2	Acrodermatitis continua
	L40.3	Pustulosis palmaris et plantaris
	L40.4	Guttate psoriasis
	L40.8	Other psoriasis
	L40.9	Psoriasis, unspecified
Rheumatoid arthritis	M05	Seropositive rheumatoid arthritis
	M06	Other rheumatoid arthritis
	M31.5	Giant cell arteritis with polymyalgia rheumatica
	M32	Systemic lupus erythematosus
	M33	Dermatopolymyositis
	M34	Systemic sclerosis
	M35.1	Other overlap syndromes
	M35.3	Polymyalgia rheumatica
	M36.0	Dermato(poly)myositis in neoplastic disease
Schizophrenia	F20	Schizophrenia
	F21	Schizotypal disorder
	F23.2	Acute schizophrenia-like psychotic disorder
	F25	Schizoaffective disorders
Severe constipation	K55.8	Other vascular disorders of intestine
	K56.0	Paralytic ileus

Condition	ICD-10 codes	Description
	K56.4	Other impaction of intestine
	K56.7	Ileus, unspecified
	K59.0	Constipation
	K63.1	Perforation of intestine (nontraumatic)
	K63.4	Enteroptosis
	K63.8	Other specified diseases of intestine
	K92.8	Other specified diseases of digestive system
Stroke or transient ischaemic attack	G45.0	Vertebro-basilar artery syndrome
	G45.1	Carotid artery syndrome (hemispheric)
	G45.2	Multiple and bilateral precerebral artery syndromes
	G45.3	Amaurosis fugax
	G45.8	Other transient cerebral ischaemic attacks and related syndromes
	G45.9	Transient cerebral ischaemic attack, unspecified
	H34.1	Central retinal artery occlusion
	I60	Subarachnoid haemorrhage
	I61	Intracerebral haemorrhage
	I63	Cerebral infarction
	I64	Stroke, not specified as haemorrhage or infarction

Note: Minor changes to the Tonelli et al. (2015) published algorithms were made. The algorithms developed by Tonelli required a combination of hospitalization, claims and outpatient codes over varying time periods. We modified this to at least one hospitalisation in the five years prior to index for all morbidities. For identifying chronic kidney disease, Tonelli supplemented the algorithm using lab data, however, to maintain consistency with other conditions, we only used administrative data. The codes for “peptic ulcer disease” referred to “peptic ulcer disease without haemorrhage or perforation”, therefore we amended the morbidity description. As specific codes for atrial fibrillation were only implemented in Scotland from April 2016 (ICD-10 version 2016), we included “atrial fibrillation and flutter” (ICD-10 code I48). No entry in ICD-10 was identified for: C46.71 (replaced with C46.7); M25.50, M25.51, M25.55, M25.57 (replaced with M25.5); K63.81 and K63.88 (replaced with K63.8), K92.80 and K92.88 (replaced with K92.8).
