# Supplementary material for: Identifying multimorbidity clusters in an unselected population of hospitalised patients
Source: Sci Rep. 2022 Mar 24;12:5134. doi: 10.1038/s41598-022-08690-3 (PMC8948299; doi:10.1038/s41598-022-08690-3)
Supplement: Supplementary file 2 — Supplementary Information 2. [file 41598_2022_8690_MOESM2_ESM.pdf]

Additional file 2. Characteristics of patient admissions with missing CHI numbers, and characteristics of patients with missing SIMD quintile or UR category

Age-sex distribution of admissions with missing CHI numbers (adults with an inpatient stay 2014)

Age	Female	Male	Total
18-20	8	17	**25**
21-30	33	111	**144**
31-40	17	86	**103**
41-50	29	88	**117**
51-60	18	84	**102**
61-70	30	47	**77**
71-80	33	29	**62**
≥81	19	13	**32**
**Total**	**187**	**475**	**662**

Patients with missing SIMD quintile and Urban-Rural category

**Characteristic**	**SIMD missing**		**U-R missing**	
	**n**	**%**	**n**	**%**
Total	***314***		***576***	
**Males, n (%)**	155	(49.4)	258	(44.8)
**Age, median (IQR)**	47	(31-62)	43	(29-60)
**Admission type, n (%)**				
**Routine**	82	(26.1)	156	(27.1)
**Emergency**	232	(73.9)	420	(72.9)
