# Supplementary figures and images for: Identifying multimorbidity clusters in an unselected population of hospitalised patients
Source: Sci Rep. 2022 Mar 24;12:5134. doi: 10.1038/s41598-022-08690-3 (PMC8948299; doi:10.1038/s41598-022-08690-3)

### Additional file 3. Assessing clustering tendency – visual inspection of data

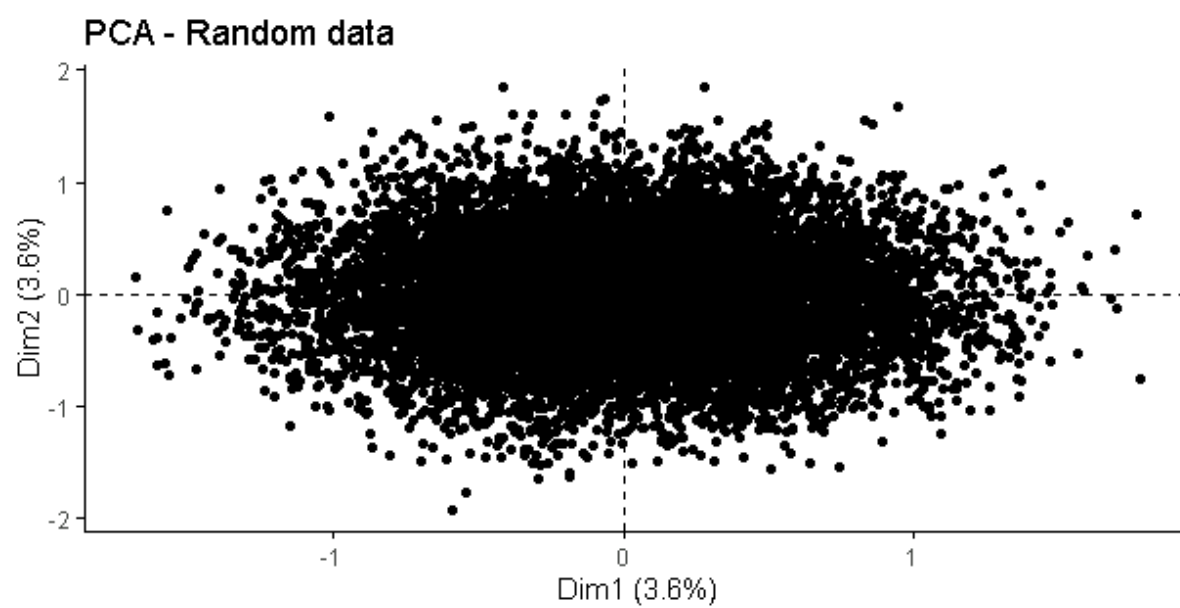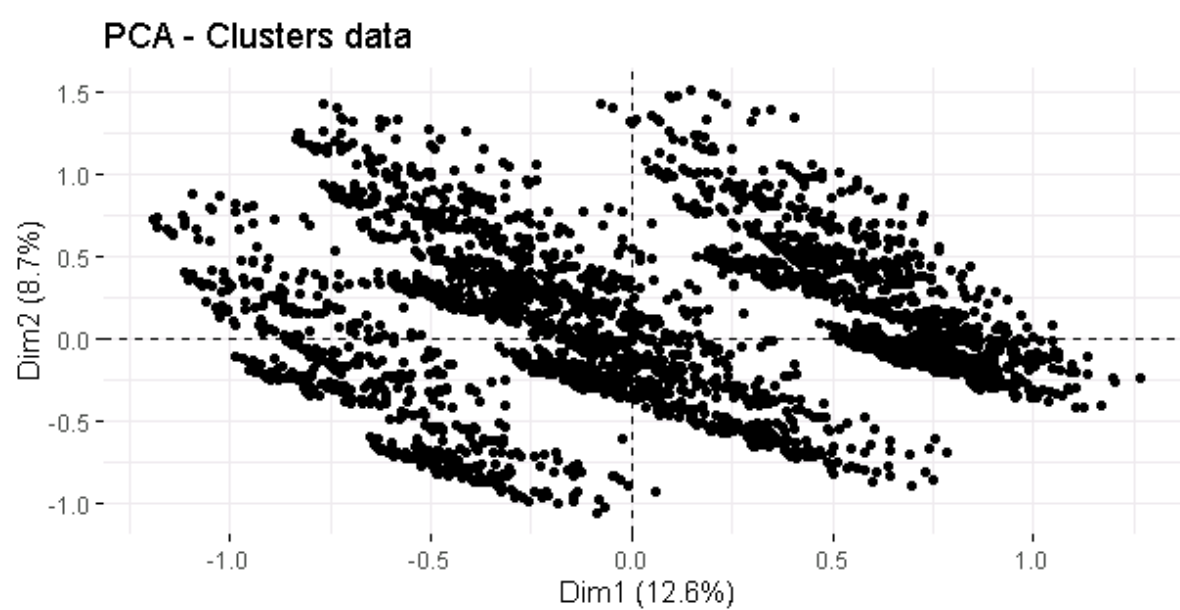

Supplement: Supplementary file 3 — Supplementary Information 3. [file 41598_2022_8690_MOESM3_ESM.pdf]
