# Supplementary material for: Identifying multimorbidity clusters in an unselected population of hospitalised patients
Source: Sci Rep. 2022 Mar 24;12:5134. doi: 10.1038/s41598-022-08690-3 (PMC8948299; doi:10.1038/s41598-022-08690-3)
Supplement: Supplementary file 4 — Supplementary Information 4. [file 41598_2022_8690_MOESM4_ESM.pdf]

Additional file 4. Transparent changes

This study was prospectively pre-registered on the Open Science Framework on 26 September 2019 (https://osf.io/qnpw2). Deviations from the pre-registered protocol were as follows:

- We planned to carry out hierarchical cluster analysis (HCA). However, HCA did not result in a clinically relevant clustering solution, as the optimal number of clusters determined was two, with the majority of patients in one cluster (both when including and excluding hypertension).

- Criteria for defining and labelling clusters of conditions was not pre-specified, as we preferred to clinically review the resulting patterns of conditions in groups of patients. We have however reported the prevalence of all conditions in each group of patients identified.

- The threshhold for excluding conditions with a high or low prevalence were not pre-specified.
