# Supplementary material for: Identifying multimorbidity clusters in an unselected population of hospitalised patients
Source: Sci Rep. 2022 Mar 24;12:5134. doi: 10.1038/s41598-022-08690-3 (PMC8948299; doi:10.1038/s41598-022-08690-3)
Supplement: Supplementary file 5 — Supplementary Information 5. [file 41598_2022_8690_MOESM5_ESM.pdf]

Additional file 5. R code

Multimorbidity clusters project - R script

Partitioning around medoids (PAM)

```
##Multimorbidity clusters project - PAM

#packages
library(cluster) # for Gower distance and PAM
library(Rtsne) # for t-SNE plot
library(ggplot2) # for visualization
library(dplyr)
library(tidyverse)
library(factoextra) # data visualization
library(NbClust) # to simultaneously compute indices for optimal number of clusters
library(rdist) # for Hamming distance


#load data
my_data <- read.csv("my_data.csv", header = T)

#check data
head(my_data, 3)
str(my_data)


##assess clustering tendency

#Visual inspection of the data

set.seed(123)

#plot my data
fviz_pca_ind(prcomp(my_data[,8:37]), title = "PCA - Clusters data",
        palette = "jco", geom = "point")

#generate random data from my_data. Added "round" to code so can use 0,1s as integers
random_df <- apply(my_data[,8:37], 2,
            function(x){round(runif(length(x), min(x), (max(x))),0)})
random_df <- as.data.frame(random_df)

#plot random_df
fviz_pca_ind(prcomp(random_df), title = "PCA - Random data",
        geom = "point", ggtheme = theme_classic())


#Hopkins

set.seed(123)

#Hopkins for random df
RandomHopkin <- get_clust_tendency(random_df, n = nrow(my_data[,8:37])-1, graph =
TRUE)
RandomHopkin$hopkins_stat # to get the Stat
```

```
#hopkins for my_data
Hopkins <- get_clust_tendency(my_data[,8:37], n = nrow(my_data[,8:37])-1, graph = TRUE)
Hopkins$hopkins_stat # to get the Stat
# Cluster analysis using Gower (equivalent to Jaccard for binary data) and PAM (2-15
clusters)

#amend condition variables to factor
my_data[,8:37] <- lapply(my_data[,8:37],factor)
str(my_data)

#Gower distance
set.seed(123)
gower_dist <- daisy(my_data[,8:37], metric = "gower")
summary(gower_dist)
gower_mat <- as.matrix(gower_dist)

# Most similar pair
my_data[
  which(gower_mat == min(gower_mat[gower_mat != min(gower_mat)]),
        arr.ind = TRUE) [1,],
  ]

# Most dissimilar pair
my_data[
  which(gower_mat == max(gower_mat[gower_mat != max(gower_mat)]),
        arr.ind = TRUE) [1,],
  ]

rm(gower_mat)

#PAM clustering

#silhouette width for many k using PAM
sil_width <- c(NA)

for (i in 2:15) {
  pam_fit <- pam(gower_dist, diss=TRUE, k=i)

  sil_width[i] <- pam_fit$silinfo$avg.width
}


# Plot silhouette width (higher is better)

plot(1:15, sil_width,
     xlab = "Number of clusters",
     ylab = "Silhouette width")
lines(1:15, sil_width)

# Characteristics of clusters

#amend simd and ur to factor (cols 4 and 5)
my_data[,4:5] <- lapply(my_data[,4:5], factor)
str(my_data)
```

```
# check if age normally distributed
hist(my_data$age)

pam_fit <- pam(gower_dist , diss = TRUE, k=10)

pam_results <- my_data[,] %>%
  dplyr::select(-PatientID) %>%
  mutate(cluster = pam_fit$clustering) %>%
  group_by (cluster) %>%
  do(the_summary = summary(.))

pam_results$the_summary

my_data$cluster = factor(pam_fit$clustering)

write.csv (my_data, "my_data_PAM_clusters.csv")


#get descriptives for toncount (counts of conditions by cluster)

#group into clusters
cluster1 <- subset(my_data, cluster == 1)
cluster2 <- subset(my_data, cluster == 2)
cluster3 <- subset(my_data, cluster == 3)
cluster4 <- subset(my_data, cluster == 4)
cluster5 <- subset(my_data, cluster == 5)
cluster6 <- subset(my_data, cluster == 6)
cluster7 <- subset(my_data, cluster == 7)
cluster8 <- subset(my_data, cluster == 8)
cluster9 <- subset(my_data, cluster == 9)
cluster10 <- subset(my_data, cluster == 10)

table(cluster1$toncount)
table(cluster2$toncount)
table(cluster3$toncount)
table(cluster4$toncount)
table(cluster5$toncount)
table(cluster6$toncount)
table(cluster7$toncount)
table(cluster8$toncount)
table(cluster9$toncount)
table(cluster10$toncount)




#visualise PAM clusters
tsne_obj <- Rtsne(gower_dist, is_distance = TRUE)

tsne_data <- tsne_obj$Y %>%
  data.frame() %>%
  setNames(c("X","Y")) %>%
  mutate(cluster = factor(pam_fit$clustering),
         name = my_data$PatientID)
```

```
names(tsne_data)[3] <- "Cluster"

write.csv (tsne_data, "plot_clusters_data.csv")

ggplot(aes(x=X,y=Y), data=tsne_data)+
  geom_point(aes(color = Cluster)) + scale_color_brewer(palette="Set3")


# validation

#silhouette plot for average silhouette width across clusters
fviz_silhouette(pam_fit, palette = "jco",
                ggtheme = theme_classic(),
                print.summary = TRUE)




###PAM with Hamming distance

#Hamming distance using pdist (rdist package)

set.seed(123)

ham_dist <- pdist(as.matrix(my_data[,8:37]), metric = "hamming")


#PAM clustering (with hamming distance)
#silhouette width for many k using PAM

sil_width <- c(NA)

for (i in 2:15) {
  pam_fit <- pam(ham_dist, diss=TRUE, k=i)

  sil_width[i] <- pam_fit$silinfo$avg.width
}


#Plot silhouette width (higher is better)

plot(1:15, sil_width,
     xlab = "Number of clusters",
     ylab = "Silhouette width")
lines(1:15, sil_width)


#Characteristics of clusters

pam_fit <- pam(ham_dist, diss = TRUE, k=10)

#amend simd and ur to factor (cols 4 and 5)
my_data[,4:5] <- lapply(my_data[,4:5], factor)

#amend condition variables to factor
```

```
my_data[,8:37] <- lapply(my_data[,8:37],factor)

pam_results <- my_data[,] %>%
  dplyr::select(-PatientID) %>%
  mutate(cluster = pam_fit$clustering) %>%
  group_by (cluster) %>%
  do(the_summary = summary(.))

pam_results$the_summary

my_data$cluster = factor(pam_fit$clustering)

write.csv (my_data, "my_data_PAM_clusters_hamming.csv")


# Visualise PAM

tsne_obj <- Rtsne(ham_dist, is_distance = TRUE)

tsne_data <- tsne_obj$Y %>%
  data.frame() %>%
  setNames(c("X","Y")) %>%
  mutate(cluster = factor(pam_fit$clustering),
         name = my_data$PatientID)

names(tsne_data)[3] <- "Cluster"

write.csv (tsne_data, "plot_clusters_data_hamming.csv")

ggplot(aes(x=X,y=Y), data=tsne_data)+
  geom_point(aes(color = Cluster)) + scale_color_brewer(palette="Set3")


#validation

#silhouette plot to get average silhouette width across clusters
fviz_silhouette(pam_fit, palette = "jco",
                ggtheme = theme_classic(),
                print.summary = TRUE)


# Cluster analysis using PAM excluding hypertension

my_data[,8:37] <- lapply(my_data[,8:37],factor)

#Gower distance (excluding hypertension)
set.seed(123)
gower_dist <- daisy(my_data[,c(8:23,25:37)], metric = "gower")
summary(gower_dist)
gower_mat <- as.matrix(gower_dist)

# Most similar pair
my_data[
  which(gower_mat == min(gower_mat[gower_mat != min(gower_mat)]),
        arr.ind = TRUE) [1,],
```

```
  ]

# Most dissimilar pair
my_data[
  which(gower_mat == max(gower_mat[gower_mat != max(gower_mat)]),
        arr.ind = TRUE) [1,],
  ]

rm(gower_mat)

#PAM clustering excluding hypertension
#silhouette width for many k using PAM

sil_width <- c(NA)

for (i in 2:15) {
  pam_fit <- pam(gower_dist, diss=TRUE, k=i)

  sil_width[i] <- pam_fit$silinfo$avg.width
}

## Plot silhouette width (higher is better)

plot(1:15, sil_width,
     xlab = "Number of clusters",
     ylab = "Silhouette width")
lines(1:15, sil_width)
# Characteristics of clusters

#amend simd and ur to factor (cols 4 and 5)
my_data[,4:5] <- lapply(my_data[,4:5], factor)
str(my_data)

pam_fit <- pam(gower_dist, diss = TRUE, k=9)

pam_results <- my_data[,] %>%
  dplyr::select(-PatientID) %>%
  mutate(cluster = pam_fit$clustering) %>%
  group_by (cluster) %>%
  do(the_summary = summary(.))

pam_results$the_summary

my_data$cluster = factor(pam_fit$clustering)

write.csv (my_data, "my_data_PAM_clusters_hyp.csv")


#Visualise PAM
tsne_obj <- Rtsne(gower_dist, is_distance = TRUE)

tsne_data <- tsne_obj$Y %>%
  data.frame() %>%
  setNames(c("X","Y")) %>%
  mutate(cluster = factor(pam_fit$clustering),
```

```
        name = my_data$PatientID)

names(tsne_data)[3] <- "Cluster"

write.csv (tsne_data, "plot_clusters_data_hyp.csv")

ggplot(aes(x=X,y=Y), data=tsne_data)+
  geom_point(aes(color = Cluster)) + scale_color_brewer(palette="Set3")


# validation

#silhouette plot to get average silhouette width across clusters
fviz_silhouette(pam_fit, palette = "jco",
                ggtheme = theme_classic(),
                print.summary = TRUE)


###PAM clustering excluding conditions with <5% prevalence (lymphoma, hepB, cirrhosis,
epilepsy, ibd, ibs, MS, Parkinsons, peptic ulcer, pvd, psoriasis, schizophrenia)


my_data[,8:37] <- lapply(my_data[,8:37],factor)

#Gower distance
set.seed(123)

#check I have identified correct columns
df <- my_data[,c(8:10,12:17,20:22,24:25,29,34,36:37)]
str(df)

gower_dist <- daisy(my_data[,c(8:10,12:17,20:22,24:25,29,34,36:37)], metric = "gower")
summary(gower_dist)
gower_mat <- as.matrix(gower_dist)

# Most similar pair
my_data[
  which(gower_mat == min(gower_mat[gower_mat != min(gower_mat)]),
        arr.ind = TRUE) [1,],
  ]

# Most dissimilar pair
my_data[
  which(gower_mat == max(gower_mat[gower_mat != max(gower_mat)]),
        arr.ind = TRUE) [1,],
  ]

rm(gower_mat)


#PAM clustering
#silhouette width for many k using PAM

sil_width <- c(NA)
```

```
for (i in 2:15) {
  pam_fit <- pam(gower_dist, diss=TRUE, k=i)

  sil_width[i] <- pam_fit$silinfo$avg.width
}


# Plot silhouette width (higher is better)

plot(1:15, sil_width,
     xlab = "Number of clusters",
     ylab = "Silhouette width")
lines(1:15, sil_width)

### Characteristics of clusters

#amend simd and ur to factor (cols 4 and 5)
my_data[,4:5] <- lapply(my_data[,4:5], factor)
str(my_data)

pam_fit <- pam(gower_dist , diss = TRUE, k=13)

pam_results <- my_data[,] %>%
  dplyr::select(-PatientID) %>%
  mutate(cluster = pam_fit$clustering) %>%
  group_by (cluster) %>%
  do(the_summary = summary(.))

pam_results$the_summary

my_data$cluster = factor(pam_fit$clustering)

write.csv (my_data, "my_data_PAM_clusters_lowprev.csv")


#visualise PAM
tsne_obj <- Rtsne(gower_dist, is_distance = TRUE)

tsne_data <- tsne_obj$Y %>%
  data.frame() %>%
  setNames(c("X","Y")) %>%
  mutate(cluster = factor(pam_fit$clustering),
         name = my_data$PatientID)

names(tsne_data)[3] <- "Cluster"

write.csv (tsne_data, "plot_clusters_data_lowprev.csv")

ggplot(aes(x=X,y=Y), data=tsne_data)+
  geom_point(aes(color = Cluster))


# validation
#silhouette plot to get average silhouette width across clusters
fviz_silhouette(pam_fit, print.summary = TRUE)
```

Agglomerative hierarchical clustering

```
##Multimorbidity clusters project – Hierarchical cluster analysis

##packages
library(cluster) # for HCA
library(ggplot2)
library(dplyr)
library(tidyverse)
library(factoextra) # data visualization
library(NbClust) # to simultaneously compute indices for optimal number of clusters


##load data
my_data <- read.csv("my_data.csv", header = T)


##check data
head(my_data, 3)
str(my_data)


##Agglomerative HCA with Gower (Gower is equivalent to Jaccard for binary data)
#amend condition variables to factor
my_data[,8:37] <- lapply(my_data[,8:37],factor)
str(my_data)

set.seed(123)

# compute Gower dissimilarity matrix
res_dist <- dist(my_data[,8:37], method = "binary") # binary method equivalent to
Gower/Jaccard equations for binary data

# display first 6 rows and columns of matrix
as.matrix(res_dist)[1:6, 1:6]

# perform HC with average method
res_hc <- hclust(d = res_dist, method = "average")

#display dendogram
fviz_dend(res_hc, cex = 0.5)
plot(res_hc)


# determine optimal number of clusters
# reload data and do not change variables to factors

res_NbClust <- NbClust(as.matrix(my_data[,8:37]),
                   distance = "binary", min.nc = 2, max.nc = 20, method = "average")
res_NbClust$All.index
res_NbClust$Best.nc
res_NbClust$All.CriticalValues
res_NbClust$Best.partition
fviz_nbclust(res_NbClust)
#2 clusters optimal
```

```
# NbClust using diss matrix specifying silhouette index
res_NbClust <- NbClust(diss = res_dist, distance = NULL, min.nc = 2, max.nc = 20,
                method = "average", index = "silhouette")
res_NbClust$All.index
res_NbClust$Best.nc
res_NbClust$All.CriticalValues
res_NbClust$Best.partition
fviz_nbclust(res_NbClust)
#2 clusters

# NbClust using complete linkage
res_NbClust <- NbClust(as.matrix(my_data[,8:37]),
                distance = "binary", min.nc = 2, max.nc = 20, method = "complete")

res_NbClust$All.index
res_NbClust$Best.nc
res_NbClust$All.CriticalValues
res_NbClust$Best.partition
fviz_nbclust(res_NbClust)
#2 clusters


##Agglomerative HCA with Gower (excluding hypertension)

# amend condition variables to factor
my_data[,8:37] <- lapply(my_data[,8:37],factor)
str(my_data)

set.seed(123)

# compute Gower dissimilarity matrix
res_dist <- dist(my_data[,c(8:23,25:37)], method = "binary")

# display first 6 rows and columns of matrix
as.matrix(res_dist)[1:6, 1:6]

# perform HC with average method
res_hc <- hclust(d = res_dist, method = "average")

grp <- cutree(res_hc, k = 20)
table(grp) #look at cluster numbers

#display dendogram
fviz_dend(res_hc, cex = 0.5)
plot(res_hc)

# determine optimal number of clusters
# reload data and do not change variables to factors

res_NbClust <- NbClust(as.matrix(my_data[,c(8:23,25:37)]),
                distance = "binary", min.nc = 2, max.nc = 20, method = "average")
res_NbClust$All.index
res_NbClust$Best.nc
res_NbClust$All.CriticalValues
res_NbClust$Best.partition
```

```
fviz_nbclust(res_NbClust)
#2 clusters

grp <- cutree(res_hc, k = 2)
table(grp) #look at cluster numbers
```
