# Supplementary material for: Identifying multimorbidity clusters in an unselected population of hospitalised patients
Source: Sci Rep. 2022 Mar 24;12:5134. doi: 10.1038/s41598-022-08690-3 (PMC8948299; doi:10.1038/s41598-022-08690-3)
Supplement: Supplementary file 6 — Supplementary Information 6. [file 41598_2022_8690_MOESM6_ESM.pdf]

Additional file 6. Sensitivity analyses

Cluster analysis using Hamming distance

Group number	1	2	3	4	5	6	7	8	9	10
**Number of patients**	**2,590**	**1,290**	**931**	**878**	**1,396**	**834**	**654**	**694**	**1,614**	**508**
**Prevalence of conditions (%)**										
Alcohol misuse (n=1,155)	6.9	6.0	**75.4**	3.4	4.1	2.0	0.9	3.2	3.4	1.8
Asthma (n=2,074)	4.7	**68.9**	20.5	12.2	11.3	20.6	8.6	9.5	14.1	17.1
Atrial fibrillation and flutter (n=2,262)	**59.0**	5.4	0.4	17.8	0.0	0.0	1.7	**71.2**	0.0	0.0
Cancer, lymphoma (n=179)	1.9	1.8	0.8	1.6	1.6	2.0	1.2	1.9	1.1	2.0
Cancer, metastatic (n=635)	2.3	0.7	0.5	1.7	2.7	0.7	**71.7**	0.9	1.7	0.4
Cancer, non-metastatic (n=1,291)	10.5	5.7	3.7	4.8	8.0	2.2	**92.0**	4.3	5.9	2.8
Chronic heart failure (n=1,470)	11.7	5.7	3.9	17.4	6.7	5.5	2.1	**84.6**	8.7	4.9
Chronic kidney disease (n=2,959)	8.2	5.2	1.2	**100.0**	**100.0**	0.0	4.0	**53.2**	0.0	0.0
Chronic pain (n=1,937)	6.5	8.4	15.7	12.8	16.8	**100.0**	10.2	8.1	13.1	0.0
Chronic pulmonary disease (excludes asthma) (n=2,115)	8.6	**68.4**	13.0	15.0	10.9	12.1	12.5	25.8	10.3	14.8
Chronic viral hepatitis B (n=16)	0.2	0.0	0.5	0.0	0.1	0.2	0.0	0.0	0.1	0.0
Cirrhosis and hepatic decompensation (n=383)	2.4	2.7	12.1	3.9	2.6	0.8	3.2	2.4	2.9	2.0
Dementia (n=586)	8.6	2.8	3.7	4.4	6.1	4.4	0.6	5.9	3.2	6.9
Depression (n=786)	3.1	3.9	**54.6**	3.5	3.9	1.4	0.5	1.6	1.5	2.4
Diabetes (n=3,071)	11.0	5.5	15.1	**100.0**	0.0	0.0	3.2	8.9	**100.0**	0.0
Epilepsy (n=432)	4.0	5.2	9.9	1.8	3.1	2.9	2.9	1.3	1.9	5.5
Hypertension (n=6,430)	**77.5**	**58.1**	6.3	**72.2**	**64.7**	**53.7**	9.9	14.7	**71.4**	**60.8**
Hypothyroidism (n=1,647)	7.7	5.5	9.6	12.0	14.7	14.9	7.8	10.2	13.8	**100.0**
Inflammatory bowel disease (n=347)	4.1	4.0	1.5	1.9	3.5	3.7	2.9	1.3	1.9	3.9
Irritable bowel syndrome (n=170)	1.5	2.2	2.4	0.6	0.9	3.6	0.6	0.4	0.9	2.0
Multiple sclerosis (n=128)	1.6	0.9	0.9	0.6	1.1	1.2	0.9	0.1	1.5	1.0
Myocardial infarction (n=881)	10.0	6.5	2.6	11.7	5.5	4.3	2.1	21.3	6.8	5.3
Parkinson's disease (n=188)	2.7	0.9	1.4	0.7	1.6	2.3	0.5	1.9	1.4	1.0
Peptic ulcer disease excluding bleeding (n=214)	2.4	1.7	3.2	1.1	1.5	2.2	0.8	1.6	1.7	1.2
Peripheral vascular disease (n=27)	0.2	0.3	0.0	0.3	0.3	0.1	0.0	0.3	0.3	0.4
Psoriasis (n=83)	0.5	0.5	1.2	0.5	1.1	1.3	0.5	0.3	0.7	1.2
Rheumatoid arthritis (n=579)	5.5	6.3	1.6	4.0	7.3	8.6	3.2	4.0	3.0	6.7
Schizophrenia (n=89)	0.4	1.0	4.0	0.2	0.6	0.6	0.3	0.1	0.4	0.6
Severe constipation (n=1,168)	11.2	10.9	11.0	9.3	10.6	12.2	13.3	7.2	6.8	11.0
Stroke or transient ischaemic attack (n=764)	12.6	5.3	4.5	5.7	5.4	4.1	2.1	6.3	5.5	3.9

Cluster analysis excluding hypertension

Group number	1	2	3	4	5	6	7	8	9
**Number of patients**	**1,778**	**995**	**1,238**	**911**	**1,698**	**908**	**1,390**	**1,819**	**652**
**Prevalence of conditions (%)**									
Alcohol misuse (n=1,155)	**58.1**	0.0	4.8	3.4	0.0	1.0	1.7	0.0	0.0
Asthma (n=2,074)	8.9	19.6	**72.1**	12.5	9.8	8.4	9.1	13.2	15.6
Atrial fibrillation and flutter (n=2,262)	4.4	9.6	5.3	20.1	17.9	10.0	**80.9**	12.1	14.9
Cancer, lymphoma (n=179)	1.9	2.0	1.7	1.5	1.5	1.3	1.5	1.0	1.8
Cancer, metastatic (n=635)	0.4	1.3	0.3	2.2	1.4	**58.8**	0.3	1.0	1.5
Cancer, non-metastatic (n=1,291)	1.9	5.2	2.8	5.2	7.2	**91.3**	2.2	5.2	7.1
Chronic heart failure (n=1,470)	2.4	4.4	2.5	19.8	9.5	2.3	**59.4**	7.6	4.0
Chronic kidney disease (n=2,959)	4.3	0.0	5.8	**100.0**	**100.0**	4.6	11.4	0.0	0.0
Chronic pain (n=1,937)	7.0	**100.0**	9.1	12.6	16.2	3.6	4.0	12.4	0.0
Chronic pulmonary disease (excludes asthma) (n=2,115)	7.3	11.7	**67.2**	15.8	12.4	12.3	21.3	10.5	12.7
Chronic viral hepatitis B (n=16)	0.6	0.2	0.0	0.0	0.1	0.0	0.1	0.1	0.0
Cirrhosis and hepatic decompensation (n=383)	10.1	0.9	2.4	3.8	2.1	2.4	1.2	2.4	1.5
Dementia (n=586)	8.4	4.7	3.2	4.5	6.5	0.9	5.5	4.0	6.3
Depression (n=786)	20.8	5.9	8.3	3.5	3.4	1.3	2.7	4.2	5.7
Diabetes (n=3,071)	8.8	0.0	5.8	**100.0**	0.0	2.8	6.3	**100.0**	0.0
Epilepsy (n=432)	9.3	2.7	5.5	1.8	2.7	2.3	1.8	1.9	4.6
Hypertension (n=6,430)	46.5	48.7	**51.5**	**69.6**	**63.1**	38.3	**60.4**	**67.4**	**55.2**
Hypothyroidism (n=1,647)	3.7	13.9	5.7	12.1	15.3	2.0	5.3	14.2	**100.0**
Inflammatory bowel disease (n=347)	5.0	3.4	4.2	2.0	3.2	2.6	1.3	1.9	3.5
Irritable bowel syndrome (n=170)	2.6	3.5	2.3	0.5	0.9	0.6	0.6	0.8	1.8
Multiple sclerosis (n=128)	2.4	1.0	1.0	0.5	0.9	0.8	0.4	1.4	0.8
Myocardial infarction (n=881)	7.3	4.1	6.1	11.6	7.2	2.9	16.0	6.9	5.1
Parkinson's disease (n=188)	3.3	2.4	1.1	0.7	1.8	0.6	1.6	1.3	0.9
Peptic ulcer disease excluding bleeding (n=214)	4.2	1.9	1.5	1.1	1.3	1.0	1.8	1.6	0.8
Peripheral vascular disease (n=27)	0.2	0.1	0.3	0.3	0.2	0.0	0.2	0.3	0.3
Psoriasis (n=83)	1.0	1.4	0.6	0.4	0.8	0.2	0.3	0.8	0.9
Rheumatoid arthritis (n=579)	5.2	8.2	6.1	3.8	6.9	2.6	4.3	3.2	4.9
Schizophrenia (n=89)	2.4	0.6	1.2	0.2	0.4	0.1	0.2	0.4	0.8
Severe constipation (n=1,168)	14.4	12.7	11.1	9.1	10.7	11.9	5.7	7.0	10.6
Stroke or transient ischaemic attack (n=764)	10.9	4.7	5.0	5.5	6.2	2.9	10.4	5.8	4.6

Cluster analysis excluding conditions with a prevalence of <5%

Group number	1	2	3	4	5	6	7	8	9	10	11	12	13
**Number of patients**	**1,443**	**755**	**1,251**	**634**	**1,231**	**1,155**	**672**	**555**	**857**	**1,188**	**543**	**449**	**656**
**Prevalence of condition (%)**													
Alcohol misuse (n=1,155)	8.5	4.5	10.2	3.9	4.2	2.0	2.5	1.1	2.5	5.0	11.8	15.4	**81.7**
Asthma (n=2,074)	0.0	11.7	**100.0**	12.3	11.9	11.2	11.9	3.4	6.9	13.6	0.0	0.0	9.5
Atrial fibrillation and flutter (n=2,262)	0.0	**100.0**	5.0	21.1	10.5	19.4	0.0	1.6	**88.6**	0.0	14.9	14.3	6.7
Cancer, lymphoma (n=179)	1.6	0.7	0.8	1.7	3.2	1.0	2.1	1.4	2.0	0.4	2.4	3.3	0.9
Cancer, metastatic (n=635)	2.5	2.0	1.0	2.1	1.1	2.9	0.9	**81.6**	1.9	1.6	1.5	1.1	0.8
Cancer, non-metastatic (n=1,291)	12.7	7.0	6.4	5.5	5.2	8.1	2.8	**93.3**	7.2	5.1	6.4	13.8	4.0
Chronic heart failure (n=1,470)	10.7	0.0	3.7	20.5	8.0	8.7	4.0	0.7	**80.0**	6.7	11.2	14.0	3.0
Chronic kidney disease (n=2,959)	0.0	0.0	0.0	**100.0**	**76.6**	**100.0**	0.0	4.7	21.8	0.0	0.0	0.0	2.1
Chronic pain (n=1,937)	0.0	10.1	17.1	13.2	13.7	13.6	**100.0**	10.1	12.0	11.8	14.0	17.4	17.1
Chronic pulmonary disease (excludes asthma) (n=2,115)	22.9	14.8	24.9	16.4	11.1	15.7	10.4	4.7	16.5	11.5	15.5	**100.0**	5.0
Chronic viral hepatitis B (n=16)	0.1	0.0	0.2	0.0	0.4	0.0	0.3	0.0	0.0	0.0	0.2	0.0	0.6
Cirrhosis and hepatic decompensation (n=383)	2.1	1.3	2.3	3.5	5.2	1.9	0.7	3.6	1.6	2.7	5.9	3.8	13.0
Dementia (n=586)	8.8	5.0	2.0	4.9	7.9	6.5	5.2	0.4	6.0	3.1	4.8	4.5	3.4
Depression (n=786)	4.5	1.5	7.1	3.6	2.5	3.3	1.9	0.5	1.2	2.1	7.9	5.3	**62.7**
Diabetes (n=3,071)	0.0	20.1	11.4	**100.0**	19.8	0.0	0.0	3.6	13.5	**100.0**	**100.0**	0.0	4.9
Epilepsy (n=432)	4.0	1.1	5.2	1.9	6.9	2.0	3.3	3.1	1.9	1.3	3.9	4.9	10.5
Hypertension (n=6,430)	**100.0**	**100.0**	27.4	**100.0**	0.0	**100.0**	**70.5**	11.9	35.1	**100.0**	0.0	0.0	10.8
Hypothyroidism (n=1,647)	19.1	11.4	14.1	11.4	18.8	15.3	15.9	8.3	13.8	11.4	18.8	12.5	9.8
Inflammatory bowel disease (n=347)	2.9	1.6	3.9	1.6	8.5	1.6	3.7	3.1	1.3	1.4	3.1	2.9	1.5
Irritable bowel syndrome (n=170)	1.2	0.7	2.8	0.5	2.7	0.4	3.1	0.7	0.8	0.7	1.3	1.1	2.9
Multiple sclerosis (n=128)	1.4	0.1	1.0	0.8	2.8	0.3	1.3	1.1	0.5	0.8	3.1	0.0	1.1
Myocardial infarction (n=881)	9.8	6.4	4.6	13.6	7.0	7.4	4.2	2.3	19.5	6.9	7.2	6.5	2.6
Parkinson's disease (n=188)	2.7	0.7	1.0	0.9	2.5	1.6	2.5	0.4	2.0	1.0	1.8	1.6	1.8
Peptic ulcer disease excluding bleeding (n=214)	2.1	1.3	0.9	1.6	2.3	1.7	2.7	0.9	1.5	1.3	2.9	2.7	4.0
Peripheral vascular disease (n=27)	0.3	0.0	0.2	0.5	0.2	0.3	0.1	0.0	0.2	0.3	0.6	0.2	0.0
Psoriasis (n=83)	0.3	0.3	1.0	0.5	1.5	0.4	0.9	0.5	0.4	0.5	1.1	1.1	1.4
Rheumatoid arthritis (n=579)	6.5	3.3	3.7	3.6	9.1	5.5	9.8	2.9	3.7	3.1	3.9	7.1	1.7
Schizophrenia (n=89)	0.4	0.0	0.7	0.3	1.0	0.3	0.6	0.4	0.0	0.3	0.9	2.9	4.4
Severe constipation (n=1,168)	10.3	5.3	9.1	9.6	17.5	9.7	13.7	14.4	6.9	5.5	10.7	10.5	11.6
Stroke or transient ischaemic attack (n=764)	10.1	12.1	3.0	6.6	6.1	7.9	4.9	2.5	9.5	6.0	4.8	5.8	4.7

Excluded from cluster analysis: lymphoma, chronic viral hepatitis, cirrhosis, irritable bowel syndrome, multiple sclerosis, Parkinson's, peptic ulcer disease, peripheral vascular disease, psoriasis, schizophrenia
